# Does Prostate Median Lobe Really Matter for GreenLight HPS Laser Photovaporization of the Prostate

**DOI:** 10.1089/cren.2018.0053

**Published:** 2018-10-01

**Authors:** Yohan Bodokh, Patrick Julien Treacy, Laetitia Imbert de la Phalecque, Matthieu Durand

**Affiliations:** ^1^ Department of Urology, Hôpital Pasteur 2, Nice Sophia-Antipolis University, Nice, France.; ^2^ Department of Urology MSSM, New York City, New York.; ^3^ INSERM, U1189, ONCO-THAI, Lille, France.

**Keywords:** BPH, laser GreenLight, US scan, photovaporization

## Abstract

***Background:*** Benign prostatic hyperplasia (BPH) is a common pathology in elderly patients, inducing lower urinary tract symptoms. The treatment of BPH is first a medical option, then a surgical treatment, either by endoscopy or open surgery. We here report a case of GreenLight HPS™ laser photovaporization (PV) with an impaired maintenance of median lobe postoperatively, unimportant on functional results.

***Case Presentation:*** A 68-year-old man presented with lower urinary tract symptoms in the last 2 years, treated first by medicine with good response. On digital rectal examination, the enlarged prostate was homogeneous and regular. International Prostate Symptom Score (IPSS) was 30/35, Incontinence Quality of Life (iQol) 6/6, and International Index of Erectile Function 5 14/25 with regular sexual activity. Transrectal ultrasound (TRUS) reported BPH of 62 g with a median lobe of 6 g protruding into the bladder. At uroflowmetry, maximum urinary flow rate (Q_max_) was 8 mL/s for 90 cc void volume and 20 cc postvoid residual. After failure of medical treatment, we offered a surgical treatment option by laser therapy using the 180W XPS GreenLight™. At 1-month follow-up, functional outcomes were improved with a Q_max_ of 11 mL/s, postvoiding residual volume 0 cc, IPSS 12/35, and iQol 2/6. At 3-month follow-up, outcomes still improved, although the TRUS reported a prostate volume of 30 g with a persistent median lobe.

***Conclusion:*** Impaired maintenance of median lobe after GreenLight laser PV does not seem to affect functional results. This case report opens the way for a new therapeutic strategy for patients according to their prostate anatomy. A randomized clinical trial could be done about surgical treatment for patient BPH according to prostate volume and anatomy.

## Introduction

Benign prostatic hyperplasia (BPH) is a common male pathology over fifty. BPH is an increase of the prostatic volume, more precisely the transitional zone. Around 80% of male present microscopic prostate hyperplasia but only 20% will present lower urinary tract symptoms, such as storages or irritating symptoms, with no correlation between volume and symptoms.^1^

The treatment of BPH is indicated only in the presence of urinary symptoms, first by medical treatment (α blockers, 5α reductase inhibitors, and herbal medicine) and then in case of complication or inefficiency of medicine by either endoscopic or open surgery.^1^ The GreenLight HPS™ laser photovaporization (PV) became one of the most used endoscopic surgeries in the treatment of BPH, being an efficient substitute to transureteral prostatic resection. The aim of this surgery is to reduce prostatic volume, with the idea of a decrease in the urinary symptoms.

We report here the case of a man who underwent GreenLight HPS laser PV procedure for a significant BHP with median lobe, confirmed by transrectal ultrasound (TRUS), with efficient postoperative functional results, even though impaired maintenance of median lobe.

## Case Report

This is the case of a 68-year-old Caucasian male, with a medical history of BPH. For 2 years, the patient has reported storage symptoms such as pollakiuria (eight times per day), nocturia (three times per night), urgency, an urgency urinary incontinence associated with urinary pain. He has also experienced additional voiding symptoms, that is, staining, intermittency, slow stream, and terminal dribble. On digital rectal examination (DRE), prostate was homogeneous, regular, with an enlarged gland. International Prostate Symptom Score (IPSS) was 30/35, Incontinence Quality of Life (iQol) 6/6, and International Index of Erectile Function 5 14/25 with regular sexual activity.

**FIG. 1.**
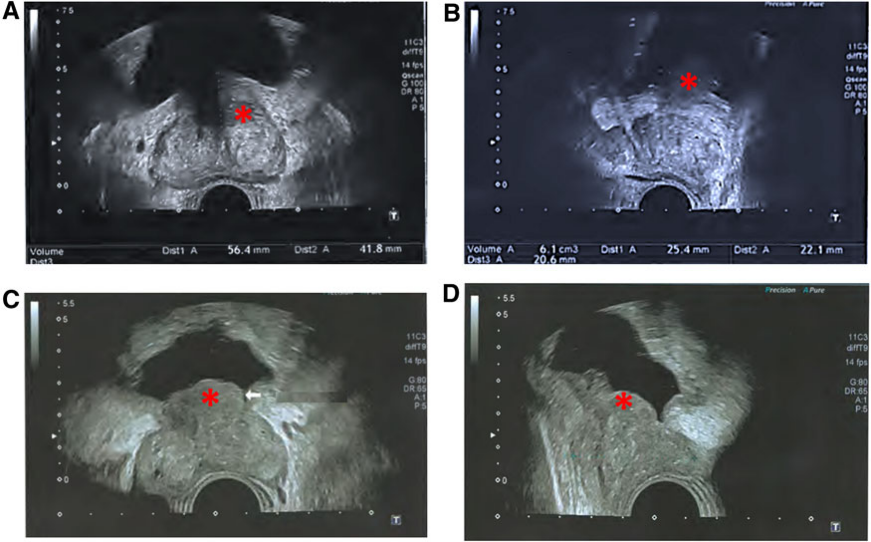
TRUS examination before **(A, B)** and after surgery **(C, D)**. This picture represents the face **(A, C)** and side **(B, D)** of prostate, on TRUS examination before GreenLight photovaporization **(A, B)** and after **(C, D)**. We find a 62 g prostate with median lobe protruding into the bladder (*) before surgery that stayed after with a major decrease of prostate volume (30 g). TRUS, transrectal ultrasound.

Prostate specific antigen (PSA) total value was 5.63 ng/cc with a ration T/L of 9.2%. The TRUS reported BPH of 62 g with a median lobe of 6 g protruding into the bladder. The postvoid residual (PVR) volume was 22 cc (Fig. 1). The blood analysis showed good renal function (clearance 100 mL/m^−1^). At uroflowmetry, maximum urinary flow rate (Q_max_) was 8 mL/s for 90 cc void volume and 20 cc PVR.

Sextant biopsies were carried out with 12 negative cores. First a medical treatment was introduced by α blockers once a day. During the follow-up, medical therapy failed overtime, with no decrease of the LUTS. He was then offered a surgical treatment option by laser therapy using the 180W XPS GreenLight™, with early catheter removal program. There was no contraindication to general anesthesia, the patient had a physical status score ASA 2, Mallampati 1.

To treat his prostate, the patient underwent a photovaporization of the prostate (PVP) under general anesthesia, using a non-morphine analgesic drug protocol to reduce the risk of acute urinary retention after early catheter removal. The cystoscopy noted a bulging prostate median lobe. A GreenLEP technique was done, using photo enucleation with one MOXY fiber par laser GreenLight™, with a total of 326 kJ delivered energy in 40 minutes. Overall surgical time was 1 hour and 10 minutes and no complication was reported. The patient was discharged the day after surgery with a medical prescription of tamsulosin LP 0.4 mg per day for 15 days.

At 1-month follow-up, functional outcomes were improved with a Q_max_ of 11 mL/s, PVR 0 cc, IPSS 12/35, iQol 2/6. At 3-month follow-up, outcomes still improved, even though TRUS reported a prostate volume of 30 g with a persistent median lobe (Fig. 1).

## Discussion

GreenLight laser PVP has been established as a minimally invasive procedure to treat patients with BPH. However, it may be difficult to achieve adequate tissue removal from a large prostate, particularly those with an enlarged median lobe.

Large prostate size, median lobes can be technically challenging during the BPH vaporization and question on their impact on vaporization treatment efficiency. Prostate gland volume is generally assessed by TRUS examination in conjunction with other parameters such as DRE and PSA. Using ultrasound (US) scan, the volume is most commonly measured using the formula, prostate volume = height ×width × length × pi/6, which is derived considering the gland as ellipsoid. There are conflicting data regarding the accuracy of this method and mostly of the relationship between the volume and the BPH assessment. Should we trust the prostatic volume measured by US? The aim of this case report was to determine the relationship between prostate gland volume and BPH diagnosis workup.

Improvement after surgery is greatest in those with the worst symptoms. Marked improvement occurs in about 93% of men with severe symptoms and in about 80% of those with moderate symptoms. The volume of the prostatic gland is not always likely to be an excellent surrogate for BPH treatment follow-up.^1^

Al-Ansari et al.^2^ showed in a comparative study between PV and endoscopic resection, a significant prostatic volume regression at 1-month after surgery, without difference between the two groups. This prostatic volume stayed stable 3 years after surgery.

Horasanli et al.^3^ in 2008 had a study involving specifically prostate volumes over 70 g resistant to medical therapy. In their study too, they highlighted a regression of prostate volume of 40% *vs* 63%, respectively, for PV and resection, but with no statistically significant difference in IPSS, iQol, or uroflowmetry decrease.

Capitan et al.^4^ demonstrated in a 2-year follow-up comparative randomized study, endoscopic resection *vs* PV, that when there was a decrease of half prostate volume, a loss of 13 points IPSS was shown, with a Q_max_ increase from 8 to 23 mL/s, without a statistical difference between the two types of surgery.

In the first study on GreenLight^®^ 180W, Bachmann et al.^5,6^ highlighted also an improvement of IPSS, iQol, and uroflowmetry, with a decrease of prostate volume of 35% after surgery and surgery time compared with 120W fiber.

In a review about BPH PV GreenLight 180W therapy, Misraï et al.^7^ reported a 50% decrease of prostate volume without differences between PV or photo enucleation, and still a decrease of IPSS, iQol, and amelioration of uroflowmetry. There was no difference regarding surgery tolerance.

Large median lobe of the prostate is likely to act as ball valve and may be a reason of medical treatment failure. It is common situation to have a prominent, large median lobe of the prostate protruding into the bladder and blocking the urine flow in a ball valve manner. In this setting, minimally invasive surgical therapy with GreenLight laser of the prostate, including the median lobe, may be recommended providing adequate relief of symptoms and long-term efficiency. As such, our patient who was not cured with an associated median lobe resection, with no consequences on functional results, introduces the question of a trigger zone.^6,7^

In a comparative study of 121 patients, comparing vaporization *vs* vaporization plus enucleation of median lobe GreenLight 120W, Kim et al.^8^ showed that there is an improvement of IPSS score without differences between two groups at 1 year after surgery, and with the same tolerance.

Prostate configuration has little effect on the efficiency of GreenLight laser PVP, so that should not be an issue in BPH workup. Indeed, a recent study compared the efficiency of GreenLight PVP depending on the prostate gland anatomy (presence or absence of median lobe). The article showed, according to IPSS iQol, Q_max_, and PVR, an improvement in the two groups without statistically significant difference between the two groups, from 1 to 36 months after surgery. These two conclusions could not incriminate the surgical technique for the results of our patient, and it can explain why we have such good results regarding the quality of life and functional outcomes in our patient.^9^

Although BPH size and other characteristics may be considered for prostate surgery, prostate median lobe did not affect recovery of urinary or sexual function. Therefore, considering the presence of median lobe in BPH vaporization is likely to be useless for management. A longer prospective study is needed.

The TRUS examination after surgery of BPH shows limited interest. The imaging might be required for the evaluation of a prostatic new growth in case of increased lower urinary tract symptoms.

## Conclusion

This case report shows the improvement of lower urinary tract symptoms despite the persistence of median lobe. This case report opens the way for a new therapeutic strategy for patients according to their prostate anatomy. A randomized clinical trial could be done about surgical treatment for patient BPH according to prostate volume and anatomy.

## Abbreviations Used

BPH: benign prostatic hyperplasia
DRE: digital rectal examination
PV: photovaporization
PVP: photovaporization of the prostate
PVR: postvoiding residual volume
Q_max_: maximum urinary flow
TRUS: transrectal ultrasound

## References

[1] HAS Guidelines Department. Diagnosis and Treatment of Benign Prostatic Hyperplasia. St Denis La Plaine, France: ANAES, 2003

[2] Al-Ansari A, Younes N, Sampige VP, Al-Rumaihi K, Ghafouri A, Gul T, Shokeir AA GreenLight HPS 120-W laser vaporization versus transurethral resection of the prostate for treatment of benign prostatic hyperplasia: A randomized clinical trial with midterm follow-up. Eur Urol 2010;58:349–355 20605316 10.1016/j.eururo.2010.05.026

[3] Horasanli K, Silay MS, Altay B, Tanriverdi O, Sarica K, Miroglu C Photoselective potassium titanyl phosphate (KTP) laser vaporization versus transurethral resection of the prostate for prostates larger than 70 mL: A short-term prospective randomized trial. Urology 2008;71:247–251 18308094 10.1016/j.urology.2007.09.017

[4] Capitán C, Blázquez C, Martin MD, Hernández V, de la Peña E, Llorente C GreenLight HPS 120-W laser vaporization versus transurethral resection of the prostate for the treatment of lower urinary tract symptoms due to benign prostatic hyperplasia: A randomized clinical trial with 2-year follow-up. Eur Urol 2011;60:734–739 21658839 10.1016/j.eururo.2011.05.043

[5] Bachmann A, Muir GH, Collins EJ, Choi BB, Tabatabaei S, Reich OM, Gómez-Sancha F, Woo HH 180-W XPS GreenLight laser therapy for benign prostate hyperplasia: Early safety, efficacy, and perioperative outcome after 201 procedures. Eur Urol 2012;61:600–607 22153927 10.1016/j.eururo.2011.11.041

[6] Bachmann A, Tubaro A, Barber N, et al. 180-W XPS GreenLight laser vaporisation versus transurethral resection of the prostate for the treatment of benign prostatic obstruction: 6-Month safety and efficacy results of a European Multicentre Randomised Trial—The GOLIATH study. Eur Urol 2014;65:931–942 24331152 10.1016/j.eururo.2013.10.040

[7] Misraï V, Rouprêt M, Guillotreau J, Bordier B, Bruyère F Greenlight^®^ photoselective vaporisation for benign prostatic hyperplasia: A systematic review. Prog Urol 2013;23:77–87 23352299 10.1016/j.purol.2012.10.013

[8] Kim KS, Choi SW, Bae WJ, et al. Efficacy of a vaporization resection of the prostate median lobe enlargement and vaporization of the prostate lateral lobe for benign prostatic hyperplasia using a 120-W GreenLight high-performance system laser: The effect on storage symptoms. Lasers Med Sci 2015;30:1387–1393 25833318 10.1007/s10103-015-1740-7

[9] Gu X, Strom K, Spaliviero M, Wong C Does prostate configuration affect the efficacy and safetyof GreenLight HPS™ laser photoselective vaporization prostatectomy (PVP)? Lasers Med Sci 2013;28:473–478 22460737 10.1007/s10103-012-1085-4

